# Comparison of two systems for long-term heart rate variability monitoring in free-living conditions - a pilot study

**DOI:** 10.1186/1475-925X-10-27

**Published:** 2011-04-12

**Authors:** Jesper Kristiansen, Mette Korshøj, Jørgen H Skotte, Tobias Jespersen, Karen Søgaard, Ole S Mortensen, Andreas Holtermann

**Affiliations:** 1 The National Research Centre for the Working Environment, Lersø Parkallé 105, DK-2100 Copenhagen, Denmark; 2 Institute of Sports Science and Clinical Biomechanics, University of Southern Denmark, Campusvej 55, DK-5230 Odense M, Denmark; 3 Department of Occupational and Environmental Health, Bispebjerg Hospital, Bispebjerg Bakke 23, DK-2400 Copenhagen NV, Denmark

**Keywords:** Autonomic nervous system, field study, Holter monitoring, method comparison

## Abstract

**Objective:**

A number of small portable systems that can measure HRV are available to address questions related to autonomic regulation in free-living subjects. However, ambulatory HRV measurements obtained through use of these systems have not previously been validated against standard clinical measurements such as Holter recordings. The objective of this study was to validate HRV obtained using a commonly used system, Actiheart, during occupational and leisure-time activities.

**Method:**

Full-day ambulatory electrocardiography (ECG) signals were recorded from 8 females simultaneously using Actiheart and Holter recorders, and signals were processed to RR-interval time series. Segments of 5-minute duration were sampled every 30 minutes, and spectral components of the heart rate variability were calculated. Actiheart and Holter values were compared using Deming regression analysis and Bland-Altman plots.

**Results:**

In total, 489 segments were available with an HRV value from both Actiheart and Holter recordings after filtering out segments with >10% interpolated beats. No systematic differences between Actiheart and Holter HRV were found. The random deviations between Actiheart and Holter were comparable to the repeatability standard deviation between consecutive Holter measurements.

**Discussion:**

The results show that Actiheart is suited as a stand-alone ambulatory method for heart rate variability monitoring during occupational and leisure-time activities.

## Background

Cardiovascular disease (CVD) is by far the leading cause of morbidity and mortality in Western societies [1]. Factors in the working environment are considered to enhance the risk of CVD [2-4]. This association is generally explained by elevated stress responses, including increased sympathetic dominance of the cardiac autonomic nervous system from a poor work environment [5,6], which in the long run enhances the risk of CVD [3].

Heart rate variability (HRV) is a well-recognized method for assessing activity of the cardiac autonomic nervous system [7,8]. HRV has been shown to be a strong predictor for mortality after acute myocardial infarction [9,10] and CVD in healthy subjects [11-13]. Therefore, HRV is regarded to be a useful non-invasive predictor for CVD.

HRV is increasingly being used for measuring the activity of the cardiac autonomic nervous system in relation to work [14]. Although HRV is influenced by physical activity [15,16], studies using ambulatory HRV monitoring during work have not controlled for physical activity [14,17]. High levels of physical activity at work are shown to enhance the risk of CVD [18,19] and CVD mortality in persons of low fitness [20,21]. Therefore, valid ambulatory monitoring of HRV during activities of free living (*i.e. *work, leisure time and sleep) is required.

Monitoring HRV in subjects during free living is challenging compared to monitoring HRV under controlled conditions. The equipment should not interfere with the activities undertaken by the subjects, including strenuous work, awkward work postures, sports activities, etc. Sleep may present another type of challenge because leads and electrodes may feel annoying and could interfere with movements during sleep. Recently, small and robust monitors have been developed for monitoring during training and exercise that enable long-term electrocardiac monitoring as well as accelerometry [22-25]. These monitors allow researchers to address questions related to physical activity at work and during leisure time that have hitherto been difficult to approach.

While the performance of different algorithms used to process this time series has been investigated and compared in a number of contexts, for example, [26-28], the reliability of the sampling of raw data, that is, the interbeat interval time series, using the small portable monitors in free-living subjects has to our knowledge not been investigated. Usually, only the timing of the heart beats is recorded in these systems. Thus, checking the electrocardiogram for noise and other artefacts is not an option. This is potentially a serious concern when collecting heart beat recordings from physically active free-living subjects.

Actiheart (CamNtech Ltd U.K.) is a small lightweight system that has been applied in a few studies for monitoring HRV [29,30]. Actiheart has been subjected to a number of validation studies; however, these studies have primarily focused on validating movement and heart rate (HR) detection. Thus, Brage et al. [23] did not find any significant difference in HR detected by Actiheart and in standard ECG recordings in 9 subjects engaged in various activities according to an exercise protocol. Also Barreira et al.[22], in a larger study encompassing 34 subjects, did not find any differences in HR estimated by Actiheart and in standard ECG recordings during standardized exercise. However, in free-living unspecified physical activity of 30 minutes duration, these authors observed that Actiheart overestimated HR by 3% compared to the reference system (Polar Vantage XL HRM, Polar Electro OY, Finland). These findings underscore the need to conduct at least part of the testing and validation under realistic conditions. Energy expenditure measurements by Actiheart have been validated under field conditions [24]. However, Actiheart HRV measurements have not previously been validated under field conditions. The objective of the present study was therefore to compare Actiheart ambulatory heart rate variability monitoring during activities of work, leisure time and sleep with HRV obtained using a state-of-the art clinical measurement (Holter monitoring). Specific aims were to compare 1) frequency domain HRV metrics obtained when using the two systems, 2) and to characterize the systematic and random deviations between HRV metrics obtained using these two techniques. For this purpose, ambulatory ECG was simultaneously measured with Actiheart and Holter monitors during work, leisure time and sleep in workers with physically demanding work (female cleaners).

## Methods

### Study population

Eight Caucasian women were recruited from a group of cleaning assistants who work more than 20 hours/week at a university hospital in Copenhagen, Denmark. Two of the subjects were diagnosed with hypertension and used prescribed antihypertensive medicine. Physical and physiological characteristics of the participants are summarized in Table 1. All participants were informed about their voluntary participation [31], and provided with a written informed consent. Ethical approval was received from the local ethics committee (H-D-2009-041).

**Table 1:** Characteristics of the participants (n = 8)

	Mean	SD	Range
Age (years)	35.50	17.36	19 - 58
Height (cm)	169.88	6.01	158 - 177
Weight (kg)	75.03	15.46	51.2 - 100.2
BMI (kg/m^2^)	25.90	4.70	20.2 - 33.5
Fat%	34.36	9.53	17 - 45
Waist circumference (cm)	88.08	13.93	72 - 106
Waist - hip ratio	0.79	0.14	0.53 - 0.98
Aerobic fitness (mL O_2_/min/kg)	27.93	4.75	19.9 - 33.7
Sleeping heart rate (beats/min.)	54.04	5.94	47 - 66
Systolic blood pressure (mmHg)	117.38	10.64	98 - 132
Diastolic blood pressure (mmHg)	78.21	8.94	64 - 87

### Study procedure

The study was performed at the participants' workplace during working hours. In the morning (during the first three hours of their workday), the participants met with the test instructor at their workplace, and the monitors (Actiheart and Holter) were firmly fixed to their chests. The same instructor placed electrodes and monitors on all participants. The recording lasted approximately 24 hours, covering work, leisure time and sleep. Six of the participants were measured twice and two participants once, yielding a total of 14 ambulatory ECG recordings available for analysis. The participants were informed to do their daily activities as usual and they received a diary in which they were asked to register their daily activities during the recording, and they also received an information sheet about the monitors and the recording.

### Instrumentation and tachograms

**Figure 1:**
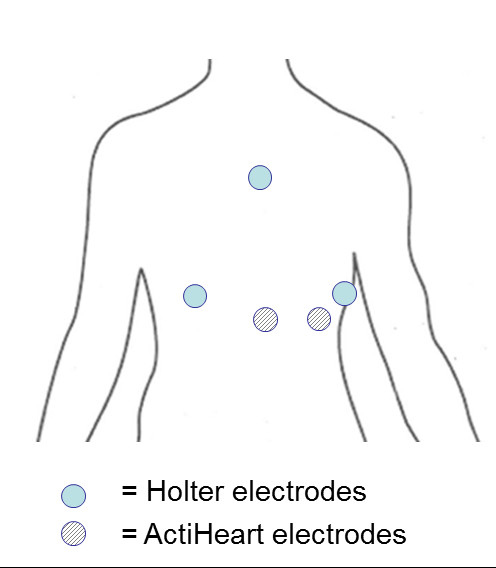
**Position of electrodes for the Holter monitor and Actiheart, respectively**.

Standard ECG Holter monitoring was made using LifeCard CF recorders (Del Mar Reynolds Medical Inc., USA), employing a 3-lead, 3-channel recording setup with a sampling frequency of 128 Hz. The three electrodes for the Holter Monitor measurement were placed on the right and left side of the chest, at the level of the 6^th ^costa and on the manubrium of sternum (Figure 1). Deviating beat forms, premature beats, pauses etc. in the ECG recordings were automatically detected by commercial software (Impresario ver. 2.8, Del Mar Reynolds Medical Inc.) and verified by visual inspection. The Impresario algorithm for detection of ectopic beats has been validated against the MIT arrhythmia database, and has a sensitivity and positive predictive power of, respectively, 99.3% and 99.7% according to information provided by the manufacturer. In order to avoid jitter in the estimation of the R-wave fiducial point, a sampling rate of at least 250 Hz is normally recommended [7]. However, satisfactory R-wave fiducial point estimation can be obtained by interpolation, such as cubic spline interpolation [7,32]. Therefore, the RR intervals were subsequently recalculated using a resampled ECG signal derived from the original ECG signal by cubic-spline interpolation from 128 Hz to 512 Hz. Although the ECG has been screened for beats deviating from sinus rhythm beats (see above) experience has shown us that further processing is necessary to prepare the RR interval series for frequency domain analyses of HRV. We therefore applied the algorithm proposed by Clifford et al. [33]. In brief, RR interval outliers were defined as RR-intervals in which both |ΔRR_n_| and |ΔRR_n+1_| are greater than δ, where ΔRR_n _is the relative change in n^th ^RR-interval. A value of δ = 0.15 was used. Finally, the RR-intervals (the tachogram) were resampled with a frequency of 4 Hz and linearly detrended.

The Actiheart recorder was fixed to the participants' upper chest by clips that fit standard ECG electrodes. The Actiheart recorder was firmly fixed to an electrode placed just below the apex of sternum while the wire running from the monitor was fixed to an electrode placed on the same horizontal level and as lateral as possible (Figure 1). This placement is associated with higher ECG amplitudes and a lower level of noise from movement artefacts, and orientated to measure acceleration along the longitudinal axis of the body [23]. Before application, the electrode placement area was prepared by cleaning the skin with 73% ethanol spirits. The procedure and electrodes used (gelled foam Delmar-Reynolds Holter/Stress) was the same for both the Actiheart and the Holter monitor recordings. In the Actiheart recorder, the analog signal was band-pass filtered (10-35 Hz), sampled with a frequency of 128 Hz, and processed by a real time QRS-detection algorithm (Actiheart User Manual, 1999). During the recording, interpolated RR intervals with a resolution of 1 ms were stored in the memory. In the recording mode employed in the present study, the raw ECG signal is not stored by the Actiheart recorder. Since the RR-intervals stored in the Actiheart recorder were not filtered beforehand, a thorough filtering was necessary before calculation of HRV for excluding abnormal RR-intervals from the tachogram. A fourstep procedure was applied, A-B-B-A, where A constitutes a similar filtering process as for the Holter recordings, and B the following method: if |ΔRR_n_| > 2 δ then d_n-1_= |RR_n-1_-M| and d_n_= |RR_n_-M| are calculated, where M is the median of the RR-intervals in the actual 5-min. segment. If d_n-1 _> d_n _then RR_n-1 _is excluded and if d_n-1 _< d_n _then RR_n _is excluded from the RR-intervals.

### Estimation of spectral components of HRV

The same mathematical algorithm was used to calculate HRV from Holter and Actiheart tachograms. In brief, the spectral components of the HRV for 5-min segments of the tachogram were estimated using Welch's averaged, modified periodogram method (Hamming window size 256 points, 50% overlap) [34]. Mean heart period (mean RR interval length) and total power (TP) were calculated for the frequency range 0.0003-0.5 Hz, low frequency power (LFP) in the range 0.04-0.15 Hz and high frequency power (HFP) in the range 0.15-0.4 Hz. Power was expressed in absolute units (ms^2^) and in normalized units (n.u.) by dividing LFP, respectively HFP, with the power in the frequency range 0.04-0.5 Hz. These power ranges correspond to the Task Force recommendations [7], except for the fact that the very high frequency range (0.4-0.5 Hz) is included in the total power frequency range by default in the Impresario software. However, since the power in the very high frequency range is very small compared to LFP and HFP power, this makes no significant difference with regard to the calculations of TP, LFn.u. and HFn.u. Finally, the ratio of power in the low frequency range to the power in the high frequency range, LF/HF, was also calculated. Only ECG recordings with ≤ 10% interpolated beats were used.

### Comparison of Actiheart and Holter recordings

We selected 5-min ECG segments starting every 30 min throughout the whole recording starting at the first available half hour, yielding 40 segments for the majority of the recordings. This sampling procedure was preferred in order to obtain approximate statistical independency of the measurements obtained in each subject. Systematic deviations between HRV metrics estimated on the basis of Actiheart and Holter recordings were investigated using Deming regression models. Contrary to ordinary least square regression, Deming regression allows errors in both variables. Deming regression is therefore preferable over least square regression in method comparison studies [35]. The random nature of the differences between Actiheart and Holter values was investigated by comparison with the repeatability standard deviation. The repeatability standard deviation expresses the variation of measurements from consecutive 5-min segments under stationary conditions. The repeatability standard deviation therefore represents a measure of the smallest differences observed between measurements. In this study, the repeatability standard deviation was estimated based on data from a previous study on biological and seasonal components of variation in HRV [36]. In brief, HRV was measured monthly in 19 persons for one year, and weekly for one month. At each time of measurement, HRV was measured in seated position for 15 min, resulting in three ECG segments of 5min duration. Applying a variance component model to the dataset allowed for estimation of the standard deviation between the HRV values measured for these three ECG segments, that is, the repeatability standard deviation.

## Results

Of a total of 582 recorded ECG segments, acceptable HRV values from both Actiheart and Holter systems were obtained in 489 segments based on the ≤10% interpolated beats criterion. More ECG segments were rejected in Actiheart (n = 69) compared to Holter (n = 28). Four ECG segments were rejected in both the Actiheart and Holter methods. Of the 513 ECG segments accepted by the Actiheart system, 24 (4.7%) were rejected in Holter analysis. An examination of the Holter ECG recordings revealed that extrasystoles and other arrhythmias were generally absent except for some normal respiratory sinus arrhythmia during sleep, and that the reason for rejection in Holter analysis was a large amount of interference from movement artefacts or electric activity from skeletal muscles or a combination of both. In the following analyses only the 489 segments with accepted HRV values from both the Actiheart and Holter systems are used.

**Figure 2:**
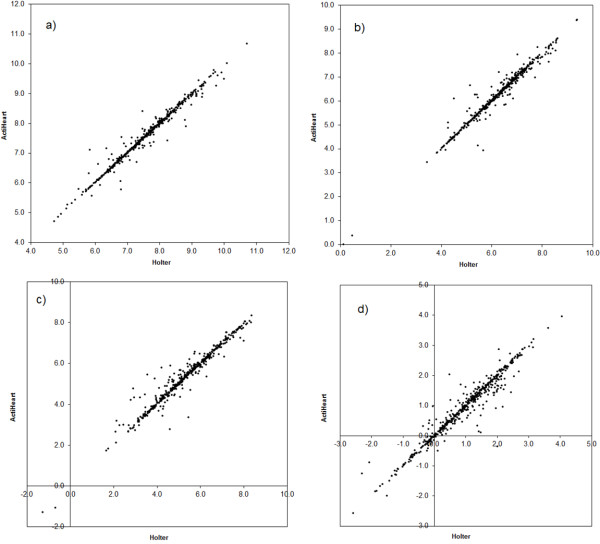
**Ambulatory HRV measurements of Actiheart versus Holter system; a) ln(TP); b) ln(LFP); c) ln(HFP); d) ln(LF/HF)**.

**Figure 3:**
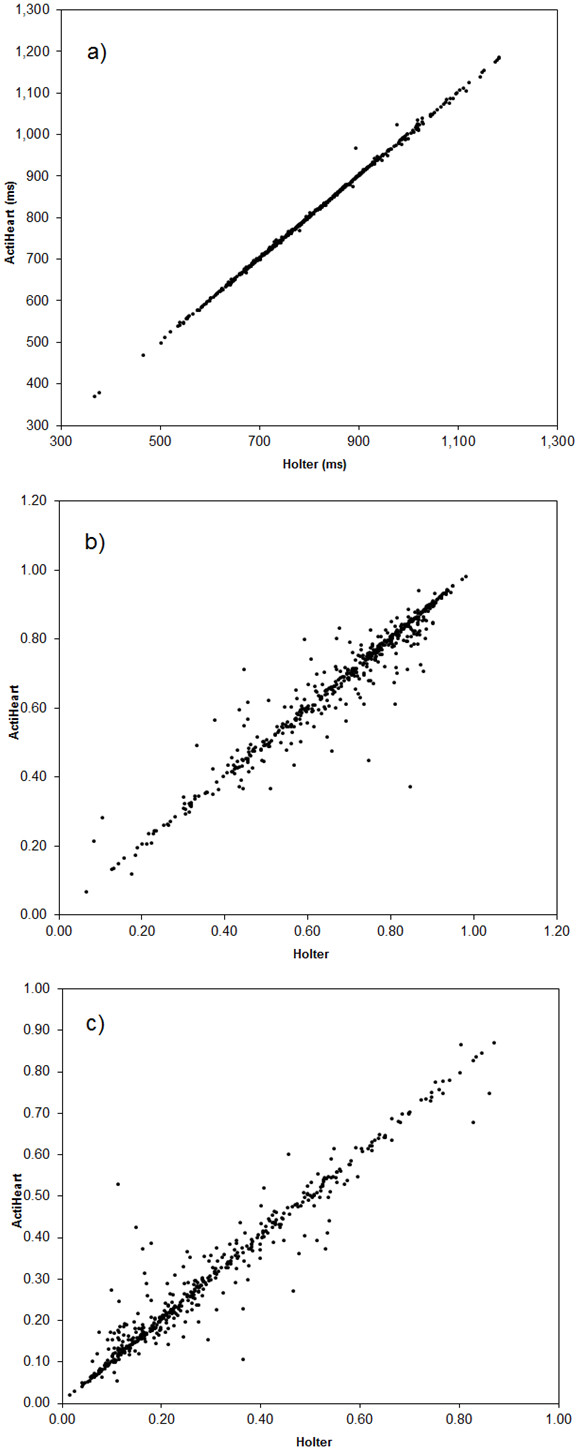
**Ambulatory HRV measurements of Actiheart versus Holter system; a) RRmean; b) LFn.u.; c) HFn.u**.

The relations between the HRV variables obtained using Actiheart and Holter are illustrated in Figures 2 and 3, and the estimated slopes and intercepts of the Deming regression analysis are presented in Table 2. The slopes are in all cases close to 1 and the deviations are in all cases ≤ 3%, and intercepts close to 0. The slopes of Actiheart versus Holter HRV are significantly smaller than 1 for HFn.u., ln(TP), ln(HFP) and ln(LF/HF). Excluding data from the two subjects using antihypertensives did not change the slopes and intercepts presented in Table 2.

**Table 2:** Slopes and intercepts (95% CI) estimated by Deming regression analyses of Actiheart versus Holter (n = 489) HRV measures.

	Slope	95%CI	Intercept	95%CI
RRmean	0.998	(0.994 - 1.001)	1.91	(-0.515 - 4.332)
LFn.u.	0.981	(0.962 - 1.001)	0.01	(-0.014 - 0.027)
HFn.u.	0.980*	(0.964 - 0.997)	0.01	(-0.002 - 0.022)
ln(TP)	0.977*	(0.962 - 0.994)	0.17*	(0.047 - 0.289)
ln(LFP)	0.995	(0.977 - 1.013)	0.03	(-0.088 - 0.166)
ln(HFP)	0.971*	(0.950 - 0.991)	0.19*	(0.061 - 0.315)
ln(LF/HF)	0.973*	(0.950 - 0.995)	0.00	(-0.043 - 0.034)

**Figure 4:**
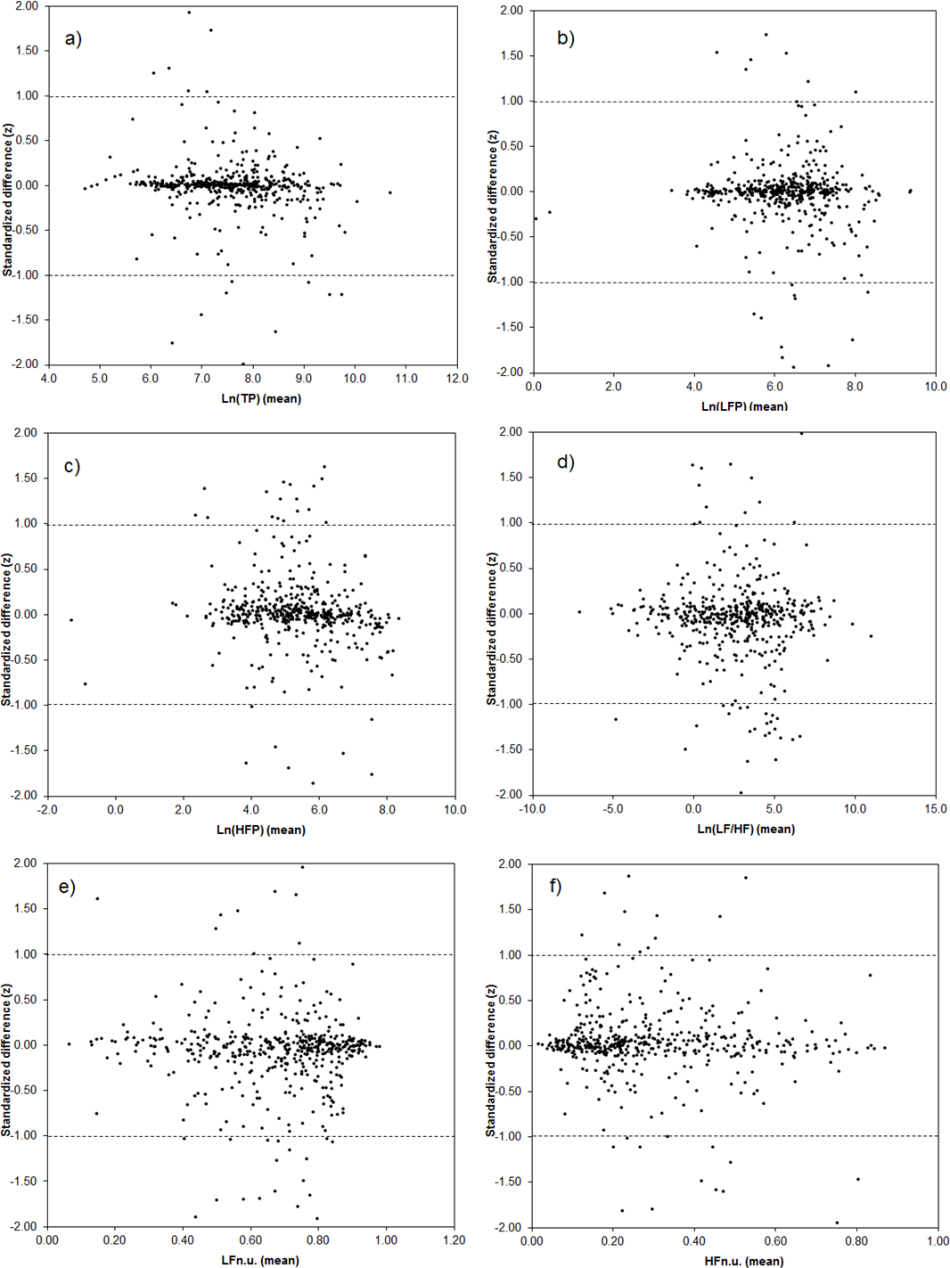
**Standardized differences of (Actiheart - Holter) HRV measures versus the mean value; a) ln(TP); b) ln(LFP); c) ln(HFP); d) ln(LF/HF); e) LFn.u.; f) HFn.u**.

Significantly different from 1 (slopes) or 0 (intercepts); * P < 0.05;

In order to investigate the magnitude of the random variation of the differences between the measurement results, standardized differences (*z) *were calculated as *z *= (Actiheart - Holter)/s_repeatability_, where s_repeatability _is the repeatability standard deviation. Figure 4 presents Bland-Altman-like plots [37] of the standardized differences versus the mean of the Actiheart and Holter HRV measurements. For all HRV measurements the deviations from the standardized difference (*z *= 0) appear to be equally distributed around 0. There is no clear tendency for the largest standardized differences to depend on the mean value. An inspection of the extreme values showed that they originated from different participants. For some of the variables, most notably for LFn.u. and HFn.u., the distribution of |*z*|-values > 1 is skewed with more extreme *z*-values at high HRV (for example, LFn.u.) or low HRV (for example, HFn.u.). However, from Figure 4 it is obvious that the skewed distribution of high or low *z*-values reflects the underlying density of HRV measurements and that the distribution is therefore not caused by a truly skewed distribution of the random errors. For example, there are very few |*z*|-values > 1 for LFn.u. > 0.5 because there are very few measurements in this range (Figure 4f). The number of standardized differences exceeding ±1 and ±2, respectively, are shown in Table 3. From 4 to10% exceed ±1while 0.8-2.5% of the values exceed ±2. This should be compared to the theoretical values for a Gaussian distribution of 32% and 5% exceeding ±1 and ±2, respectively.

**Table 3:** Number of ECG time segments for which the standardized difference between Actiheart and Holter HRV estimates (in units of repeatability standard deviations, *z*) exceeds 1 and 2, respectively.

HRV metric	*s *(repeatability)	Number of segments z>1	Percentage of total^a^	Number of segments z>2	Percentage of total^a^
ln(TP)	0.41	19	3.9%	4	0.8%
ln(LFP)	0.39	28	5.7%	10	2.0%
ln(HFP)	0.50	37	7.6%	12	2.5%
ln(LF/HF)	0.41	47	9.6%	12	2.5%
LFn.u.	0.078	39	8.0%	12	2.5%
HFn.u.	0.078	32	6.5%	8	1.6%

^a ^Total n = 489 ECG segments

**Figure 5:**
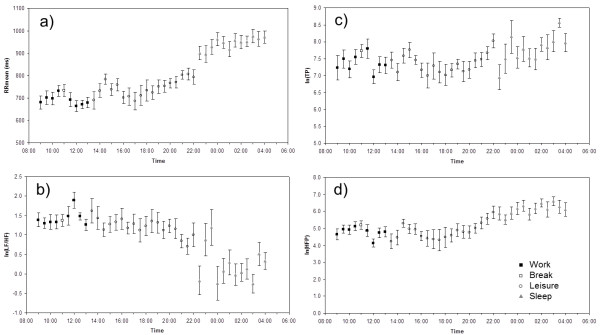
**Mean heart period (RRmean) and heart rate variability indices of 8 cleaning workers during a workday**. a) RR_mean_, b) ln(LF/HF), c) ln(TP) and d) ln(HFP) derived from 5 min recordings sampled every 30 min. Measurements are categorized according to work period (9:00 - 13:00), break during work (11:00), leisure period (13:30 - 22:00) and sleep periods (22:30 - 04:30). Error bars indicate standard error of the mean.

Lastly, Figure 5 presents data for the mean heart period and selected HRV measurements representing the total HRV (ln(TP)), the parasympathetic modulation of the autonomic cardiac regulation (ln(HFP)) and the autonomic balance (ln(LF/HF)) in the eight cleaning workers during a whole workday. Statistical analyses showed differences in heart period (lower heart rate) in the order sleep>leisure time>work (and break during work) (one-way ANOVA, P < 0.001). With regard to the HRV measurements, sleep was associated with parasympathetic dominance (higher total HRV and high frequency power, and lower ln(LF/HF)) (all P < 0.01), while no significant differences were observed during work and leisure periods.

## Discussion

Actiheart is a validated system for estimating energy expenditure based on electrocardiography (ECG) and accelerometry sensors [22-24]. The system is similar to other small portable systems used for shorter activities, e.g. exercise and training, in that the ECG cannot be stored, except for short recordings. It is therefore possible that noise, unchecked artefacts and non-normal beats, the latter well-known to significantly affect short-term HRV [38], might lower the quality of the HRV measurements obtained by the Actiheart recorder. In addition, Actiheart is a 2-lead system making it more vulnerable to electrode problems than systems with more leads. This problem could be more pronounced when measuring free-living subjects during physical activities at work or during leisure time.

In this study, we compared HRV measurements obtained through ambulatory Actiheart and Holter monitoring during an entire day. Since it is well known that different spectral analysis methods can produce different results [28,34], the tachograms from both Actiheart and Holter underwent the same spectral analysis. Likewise, the electrodes were attached by the same person to avoid influence from intertechnician effects. Differences in HRV must therefore be attributed to differences in the recorded ECG signal, different processing procedures by hardware and embedded software, as well as slightly different filtering procedures applied to the raw tachograms prior to spectral analysis.

Overall, no marked systematic differences between HRV measurements obtained by Actiheart and Holter were observed. The regression of Actiheart versus Holter values of HFn.u., ln(TP), ln(HFP) and ln(LF/HF) yielded slopes significantly different from 1. However, the differences were within a 3%-range, and therefore considered to have no practical relevance since the relative within-subject standard deviations of ln(TP), HFn.u., ln(HFP) and ln(LF/HF) are 6%, 28%, 14% and 77%, respectively [36]. Therefore, although the Deming regression analyses indicate small yet significant systematic differences between the Actiheart and Holter for some HRV measurements, the differences are too small compared to the individual differences to warrant that a correction factor be applied.

The investigation of the distribution of differences between Actiheart and Holter indicated that the differences seemed to be randomly distributed with a spread not exceeding that expected from the normal repeatability of measurements using the same method. The most extreme deviations between the measurements came from different participants, and this supports the interpretation that the differences are caused by random variations.

Some differences in the processing of the recorded ECG were unavoidable because different instruments were used. Additional processing of the RR interval series obtained from Actiheart was deemed necessary in order to obtain RR interval series of high quality. The procedures for processing ECG and RR interval time series in Holter and Actiheart are part of our standard protocol when using these instruments. Therefore, although it is possible that differences in processing of the ECG and RR interval time series can explain some of the variation between Actiheart and Holter HRV measurements, a comparison of Actiheart and Holter without applying these procotols would be less relevant. Furthermore, in view of the accordance between Actiheart and Holter, one can conclude that the contribution to the variation from differences in filtering procedures is likely to be of little importance.

These conclusions pertain to the HRV values accepted by both Actiheart and Holter measurements. A small number of measurements (n = 24, 4.7%) were accepted by Actiheart, but not by Holter. Although this proportion is small, it nevertheless suggests that as many as 5% of the Actiheart values could be erroneous. However, the signal in the rejected Holter segments was heavily disturbed by interference from electric signals from other muscles or movement artefacts, which indicates that the underlying "true" heart beat pattern was a normal sinus rhythm with no more aberrant beats than normal. However, as raw ECG data from the Actiheart are not available because they are immediately processed to yield RR-intervals without being stored in the memory, we are not able to check if Actiheart perhaps 'missed' these massive electric disturbances. Having said that, because the Actiheart electrodes are placed farther from the arms, the Actiheart electrodes may be better protected under conditions with intense movements in the upper body, and it is therefore possible that Actiheart signals are of good quality in situations where the Holter signal is unstable. Although we cannot provide definitive evidence to support this interpretation, there is no evidence to suggest that HRV values are in error with regard to these segments.

Lastly, a relatively large proportion (11.9%) of Actiheart segments were rejected, while in Holter analysis, only 5.5% were rejected. That some measurements are rejected in ambulatory monitoring due to movement artefacts and other interferences as described above is not surprising. The reason that more segments are rejected in Actiheart than in Holter might be problems caused by sweat. During the recordings, it was seen that the electrodes were not resistant to a great amount of sweat, which in particular was a problem with the electrodes for the Actiheart recordings because of their position the lower part of the chest. In a situation where one electrode does not have sufficient skin contact, the Actiheart using only two electrodes will have insufficient data sampling; the Holter monitor uses three electrodes and therefore does have this problem. Changing the positions of the electrodes may therefore reduce the loss of data observed in the present study.

The data obtained from the eight cleaners during a workday and night demonstrates the feasibility of use of the Actiheart system to study work and leisure time cardiac autonomic activity. The HRV results in Figure 5 indicating an autonomic balance of more extensive parasympathetic modulation during sleep compared to the wake state (work, leisure time) are in accordance with previous findings [36,39]. Interestingly, in a recent study Hautala et al. [40] studied the association between HRV and physical activity levels, expressed as mean metabolic equivalents (MET) for 30-min blocks, in free-living conditions. They found that physical activity was significantly associated with HR and negatively associated with short-term index of HRV and sample entropy, a measure of the complexity of the HRV. Details of the nature of the physical activity levels were not given.

In conclusion, good agreement was observed between spectral components of HRV from ambulatory short-term ECG recordings by the Actiheart and Holter systems. Although more HRV measurements were rejected in Actiheart compared to Holter monitoring, this difference was probably caused by technical problems and does not indicate a problem with Actiheart as such. Thus, Actiheart seems suitable for measurement of HRV during occupational and leisure-time activities. Additional studies including more subjects of both genders in different occupations and leisure-time activities are warranted in order to further explore the utility of Actiheart for measuring HRV in free-living subjects.

## Abbreviations

**CVD**: cardiovascular disease; **HR**: heart rate; **HRV**: heart rate variability; **ECG**: electrocardiogram; **RR**: heart interbeat interval; **TP**: total power in RR-time series power spectrum; **LFP**: low frequency power in RR-time series power spectrum; **HFP**: high frequency power in RR-time series power spectrum; **LF/HF**: ratio between LFP and HFP.

## Competing interests

The authors declare that they have no competing interests.

## Authors' contributions

JK analysed and interpreted the data, drafted and revised the manuscript. MK conceived and designed the study, acquired the data, and participated in interpretation and discussion of data. JHS analysed and interpreted the data. TJ contributed to the design of the study and acquisition of data; and participated in interpretation and discussion of data. KS conceived and designed the study, and contributed with interpretation and discussion of data. OSM contributed with interpretation and discussion of data. AH conceived and designed the study, and participated in interpretation and discussion of data. All authors contributed to draft and revise the manuscript, and all authors have read and approved the final version of the manuscript.
